# Global cognitive function and processing speed are associated with gait and balance dysfunction in Parkinson’s disease

**DOI:** 10.1186/s12984-016-0205-y

**Published:** 2016-10-28

**Authors:** Gian Pal, Joan O’Keefe, Erin Robertson-Dick, Bryan Bernard, Sharlet Anderson, Deborah Hall

**Affiliations:** 1 Department of Neurological Sciences, Rush University, Chicago, IL USA; 2 Department of Anatomy and Cell Biology, Rush University, Chicago, IL USA; 3 Rush University Medical Center, 1725 West Harrison Street, Suite 755, Chicago, IL 60612 USA

**Keywords:** Parkinson’s disease, Cognition, Processing speed, Gait, Turning

## Abstract

**Background:**

Our primary objective was to determine the relationship between global cognitive function and specific domains of gait and balance in a cohort of Parkinson’s disease (PD) subjects. In a secondary analysis, we determined whether specific cognitive domains correlated with gait and balance performance.

**Methods:**

Fourteen PD subjects (mean age 61.1 ± 7.8 years) were recruited from the Rush University Medical Center Movement Disorders clinic. Subjects underwent clinical assessment using the motor subsection of the Unified Parkinson’s Disease Rating Scale (UPDRS) followed by quantitative gait and balance assessments using the APDM Mobility Lab™ system (Mobility Lab, APDM Inc., Portland, OR). Subjects completed global cognitive testing using the Mattis Dementia Rating Scale (MDRS) as well as domain specific cognitive measures. Spearman’s rho was used to assess correlations between cognitive measures and gait and balance function, with False Discovery Rate (FDR) correction for multiple comparisons.

**Results:**

Global cognitive function had the strongest correlation with stride velocity (*r* = 0.816, *p* = 0.001), turn duration (*r* = −0.806, *p* = 0.001), number of steps to turn (*r* = −0.830, *p* = 0.001), and mean velocity of postural sway in the medio-lateral direction (*r* = −0.726, *p* = 0.005). A significant correlation was found between processing speed and two turning measures (turn duration, *r* = −0.884, *p* = 0.001; number of steps to turn, *r* = −0.954, *p* < 0.001), but no other associations were found between specific cognitive domains and gait domains.

**Conclusions:**

This pilot study provides preliminary data regarding the association between global cognitive function and pace-related measures of gait, turning, and postural sway. Furthermore, reduced processing speed was found to be associated with difficulty in performing turns.

## Background

Difficulties with gait and balance in Parkinson’s disease (PD) increase the risk of falls, institutionalization, and death [1–3]. Research studies have established that safe ambulation requires cortical input from areas involved with higher cognitive function [4, 5]. Cognitive impairment, especially prefrontal lobe dysfunction, has been linked to motor disability in PD but these relationships remain unclear [5–7].

Clinical rating scales, such as the Unified Parkinson’s disease Rating Scale (UPDRS) [8], and measures derived from such scales, such as the postural instability/gait disturbance (PIGD) score, have been used to examine the relationship between cognitive and motor impairment [9]. However, these clinical scales are subject to rater judgment and provide only limited detail because the ordinal fixed scores (0–4) force raters into a limited number of item choices without the ability to quantify disease impairment or disability as continuous measures. Thus far, these clinical scales have had limited ability to elucidate the relationship between cognition and gait and balance [10].

Body-worn inertial sensors can provide a multitude of continuous, objective measures of gait and balance [11–13]. Wearable technology allows for measurement and replication of relationships between gait and cognition without the confounding factors of heightened attentional load and observer effect seen in the laboratory [14]. Moreover, these quantitative measures can be categorized into specific domains of gait and balance for analysis [11]. In this pilot project, we used the APDM Mobility Lab™ (Mobility Lab, APDM Inc., Portland, OR), a valid and reliable measurement tool of gait and balance [15–17], to examine motor function in a small cohort of PD subjects. Our primary objective was to determine whether global cognitive function correlated with specific domains of gait and balance. Through a secondary analysis, we assessed whether specific cognitive domains correlated with gait and balance performance.

## Methods

This cross-sectional study involved PD subjects recruited from the Rush University Medical Center (RUMC) Movement Disorders clinic. The study proposal and consent were approved by the Institutional Review Board of Rush University and met standards for ethical human research. All PD patients met the UK Parkinson’s disease society brain bank clinical diagnostic criteria [18] and the diagnosis of PD was confirmed by a movement disorders neurologist through personal interview, medical history, physical examination, and chart review.

Clinical and demographic data included age at time of enrollment, age of onset, disease duration, height, and weight. Disease severity was assessed for each subject using the motor subscale of the UPDRS (UPDRS-III) [8] and Hoehn and Yahr (H&Y) [19] staging in the ON medication condition. PIGD scores were calculated by using the arithmetic sum of items 13, 14, 15, 29, 30 of the UPDRS [20].

The instrumented walk (i-WALK) and instrumented sway (i-SWAY) test protocols [21, 22] were performed using the commercially available APDM Mobility Lab™ six inertial sensor system (Mobility Lab, APDM Inc., Portland, OR), a sensitive, valid and reliable measurement tool of gait and balance in the PD population [15, 17]. The sensors were attached 4 cm above each malleolus, at the dorsum of the wrists, on the lumbar trunk at the level of L5, and on the upper trunk 2 cm below the sternal notch. The i-WALK protocol consisted of the subject walking back and forth continuously between two points 25 ft apart for a period of two minutes while in the ON medication condition. All subjects performed the i-WALK without an assistive device and the mean value for each gait parameter was calculated. A total of 23 measures were computed and categorized into four domains: pace, arm and trunk movement, dynamic stability, and turning (Appendix) [23]. Subjects then completed the i-SWAY, during which they stood still with their hands across their chest and their feet shoulder width apart or together and their eyes opened or closed. The protocol consisted of measuring balance with the feet together, eyes closed, to maximize the sensitivity of the balance testing. All assessments were completed in the ON medication condition.

After completing the i-WALK and i-SWAY, subjects rested for approximately 1–2 h. Then, a neuropsychologist administered the Mattis Dementia Rating Scale (MDRS) [24, 25], a test of global cognitive function. Subjects also completed neuropsychological measures for the following domains: verbal fluency, verbal memory (immediate and delayed), processing speed, working memory, and executive function (See Table 1).

**Table 1:** Neuropsychological battery: cognitive domains and corresponding tests

Domain	Tests
Verbal fluency	Controlled Oral Word Association Test (COWAT) [51]
Verbal Memory – Immediate	Rey Auditory Verbal Learning Test (RAVLT) - Trial 5 [52, 53]
Verbal Memory – Delayed	Rey Auditory Verbal Learning Test (RAVLT) - Delayed Recall [52, 53]
Processing speed	Symbol Digit Modalities Test (SDMT) [54]
Working memory	Auditory Consonant Trigrams (ACT) [55, 56]
Executive function	Tower of London (TOL) [57]

SPSS version 16.0 was used for statistical analysis. Spearman’s rho (*r*) was used to assess correlations between MDRS scores and measures of gait and balance, as well as to assess correlations between the domain specific cognitive tests and gait and balance measures. Raw scores from each neuropsychological test were converted to *z* scores. Multiple comparisons were accounted for by using a false discovery rate (FDR) adjustment [26] α = 0.05 [20]. FDR was considered more appropriate than the more conservative corrections, such as Bonferonni, as a large number of associations were under investigation [26, 27].

## Results

Fourteen subjects with PD were included in the analysis (Table 2). Subjects had a mean age of 61.1 ± 6.1 years and disease duration of 12.7 ± 6.2 years. Mean motor UPDRS was 21.5 ± 9.2 (ON condition) indicating that subjects had mild to moderate disease severity. The mean MDRS Total score was 132.2 ± 8.3, indicating that subjects varied between largely intact cognition and mild cognitive impairment. The MDRS Total score had the strongest correlation with pace-related measures of gait, turning, and postural sway in the ON condition (Table 3). Significant correlations were found between MDRS Total scores and stride velocity (*r* = 0.816, *p* = 0.001), turn duration (*r* = −0.806, *p* = 0.001), and number of steps to turn (*r* = −0.830, *p* = 0.001). MDRS Total scores correlated with one balance measure, mean sway velocity in the medio-lateral direction (*r* = −0.726, *p* = 0.005). MDRS Total scores had a modest correlation with motor UPDRS scores in the ON condition (*r* = −0.568, *p* = 0.043). There was no significant correlation between MDRS Total scores and PIGD scores (*p* = 0.061). Lastly, an association was found between processing speed, as measured by the SDMT, and two measures from the gait turning domain (turn duration and number of steps to turn), but no other cognitive domain had significant correlations with any gait domains (Table 4).

**Table 2:** Participant Characteristics

	Mean (SD)
n	14
Age, years	61.1 (7.8)
Age of onset, years	48.5 (10.9)
Height, m	1.72 (0.14)
Weight, kg	82.5 (23.1)
Men/Women %	66.5/33.5
Disease duration, years	12.7 (6.2)
H & Y	2.3 (0.6)
Motor UPDRS	21.5 (9.2)
PIGD	2.7 (3.4)
MDRS	132.2 (8.3)

*H & Y* Hoehn and Yahr, *UPDRS* Unified Parkinson’s disease rating scale, *PIGD* postural instability/gait disturbance score, *MDRS* Mattis dementia rating scale

**Table 3:** Correlation of Gait and Balance Domains with Global Cognitive Function^a^

	MDRS	
	*r*	*p*
Gait – Pace		
Gait cycle time, sec	−0.613	0.034
Cadence, steps/min	0.627	0.029
Stride velocity, % height/sec	**0.816**	**0.001**
Stride length, % height	0.687	0.014
RoM leg, degrees	0.687	0.014
Gait – Arm & trunk Movement		
Arm peak velocity, deg/s	0.637	0.026
Arm swing RoM, deg	0.007	0.983
Asymmetry arm swing RoM, %	−0.193	0.549
ROM trunk frontal plane, deg	0.455	0.137
RoM trunk sagittal plane, deg	0.235	0.463
RoM trunk horizontal plane, deg	0.312	0.324
Gait – Dynamic Stability		
Double support time, % of gait cycle	−0.504	0.094
Stance time, % of gait cycle	−0.504	0.094
Turning		
Peak velocity, deg/s	0.682	0.010
Duration, sec	**−0.806**	**0.001**
Mean step time, sec	−0.354	0.259
Number of steps, n	**−0.830**	**0.001**
Postural sway		
Sway RMS AP, m/s^2^	−0.558	0.047
Sway RMS ML, m/s^2^	−0.644	0.018
Mean velocity AP, m/s	−0.074	0.809
Mean velocity ML, m/s	**−0.726**	**0.005**
Centroidal frequency AP, Hz	−0.297	0.324
Centroidal frequency ML, Hz	−0.242	0.426

^a^Bolded measures indicate measures that remained significant after false discovery rate (FDR) correction

*MDRS* Mattis dementia rating scale, *ROM* range of motion, *RMS* root mean square of acceleration time series, *AP* anteroposterior, *ML* mediolateral, centroidal frequency (variability of acceleration traces power ranging from 0 to 1)

**Table 4:** Correlation of Gait and Balance Domains with Specific Cognitive Domains^a^

	Language		Verbal Memory – Immediate		Verbal Memory – Delayed		Processing Speed		Working Memory		Executive Function	
	COWAT [51]		RAVLT Trial 5 [52, 53]		RAVLT Delayed Recall [52, 53]		SDMT [54]		ACT [55, 56]		TOL [57]	
	*r*	*p*	*r*	*p*	*r*	*p*	*r*	*p*	*r*	*p*	*r*	*p*
Gait – pace												
Gait cycle time, sec	−0.624	0.040	−0.402	0.221	−0.291	0.385	−0.573	0.083	−0.219	0.571	0.256	0.507
Cadence, steps/min	0.574	0.065	0.420	0.198	0.291	0.385	0.530	0.115	0.310	0.416	−0.183	0.638
Stride velocity, %h/s	0.492	0.124	0.525	0.097	0.517	0.103	0.744	0.014	0.529	0.143	0.146	0.708
Stride length, %h	0.091	0.790	0.388	0.238	0.467	0.148	0.585	0.075	0.785	0.012	0.602	0.086
RoM leg, deg	0.091	0.790	0.388	0.238	0.467	0.148	0.585	0.075	0.785	0.012	0.602	0.086
Gait – arm & trunk Movement												
Arm peak velocity, deg/s	0.374	0.258	0.370	0.263	0.342	0.304	0.744	0.014	0.347	0.360	0.091	0.815
Arm swing RoM, deg	−0.469	0.145	−0.498	0.119	−0.199	0.558	0.085	0.815	−0.183	0.638	−0.018	0.963
Asymmetry arm swing RoM, %	−0.542	0.085	−0.438	0.177	−0.231	0.494	−0.311	0.382	−0.237	0.539	−0.146	0.708
ROM trunk frontal plane, deg	−0.287	0.392	0.128	0.708	0.217	0.521	0.335	0.343	0.639	0.064	0.475	0.197
RoM trunk sagittal plane, deg	0.082	0.811	−0.096	0.779	0.000	1.000	0.598	0.068	0.347	0.360	0.110	0.779
RoM trunk horizontal plane, deg	−0.369	0.264	−0.233	0.491	−0.037	0.914	0.024	0.947	0.091	0.815	0.183	0.638
Gait – Dynamic Stability												
Double support time, % of gait cycle	−0.292	0.384	−0.324	0.331	−0.393	0.232	−0.378	0.281	−0.548	0.127	−0.420	0.260
Stance time, % of gait cycle	−0.292	0.384	−0.324	0.331	−0.393	0.232	−0.378	0.281	−0.548	0.127	−0.420	0.260
Turning												
Peak velocity, deg/s	0.273	0.416	0.548	0.065	0.542	0.069	0.732	0.016	0.649	0.042	0.389	0.266
Duration, sec	−0.597	0.053	−0.643	0.024	−0.712	0.009	**−0.884**	**0.001**	−0.621	0.055	−0.362	0.304
Mean step time, sec	−0.433	0.184	−0.119	0.728	0.032	0.925	−0.354	0.316	−0.164	0.673	0.347	0.360
Number of steps, n	−0.409	0.212	−0.700	0.016	−0.400	0.198	**−0.954**	**<0.001**	−0.803	0.009	−0.566	0.112
Postural sway												
Sway RMS AP, m/s^2^	−0.410	0.210	−0.366	0.243	−0.400	0.198	−0.220	0.542	−0.382	0.276	−0.464	0.176
Sway RMS ML, m/s^2^	−0.255	0.449	−0.443	0.149	−0.556	0.061	−0.122	0.737	−0.580	0.079	−0.580	0.079
Mean velocity AP, m/s	−0.059	0.863	−0.460	0.132	−0.025	0.939	−0.085	0.815	0.096	0.793	0.096	0.793
Mean velocity ML, m/s	−0.410	0.210	−0.633	0.027	−0.609	0.036	−0.256	0.475	−0.574	0.083	−0.437	0.207
Centroidal frequency AP, Hz	−0.027	0.936	−0.341	0.278	−0.513	0.088	0.061	0.867	−0.615	0.059	−0.737	0.015
Centroidal frequency ML, Hz	0.077	0.821	−0.429	0.164	−0.517	0.085	0.091	0.802	−0.362	0.304	−0.608	0.062

^a^Bolded measures indicate measures that remained significant after False Discovery Rate (FDR) correction

Controlled Oral Word Association Test [51], Rey Auditory Verbal Learning Test (RAVLT) [52, 53], Symbol Digit Modalities Test (SDMT) [54], Auditory Consonant Trigrams (ACT) [55, 56], Tower of London (TOL) [57]

### False discovery rate (FDR)

A total of 161 correlations were performed. After FDR correction, 26 significant associations (Spearman’s rho > 0.2, *p* < 0.05) was reduced to six significant associations (bolded in Tables 1 and 2).

## Discussion

This pilot study adds to the body of evidence [5, 7, 28–30] that lower global cognitive function is correlated with worse performance in different domains of gait and balance. Importantly, this is the first study to demonstrate the correlation between reduced processing speed and impaired turning. Though this is a small pilot study, the validity of our results are strengthened by the strong reliability of the APDM system ([15–17, 31]. Salarian et al. [31] demonstrated that the APDM Mobility lab system has high test-retest reliability for turn duration (*ρ* = 0.89) and good reliability for the number of steps to turn (*ρ* = 0.75).

Prior studies [32] have shown that executive function plays a role in turning, but the domain of executive function may be too broad to provide meaningful results in a clinical or research context [33]. Processing speed is a more precise construct as it is a basic cognitive process that subserves higher-order cognitive domains such as executive function [34]. The mechanism of reduced processing speed in PD has been explored in several studies. Jokinen et al. [35] used [^18^F]fluorodopa (Fdopa) Positron Emmision Tomography (PET) to demonstrate that reduced Fdopa uptake in the anterior cingulate gyrus, thalamus, and caudate nucleus was related to decreased processing speed. Likewise, when examining the predictive role of gray and white matter fractional anisotropy on processing speed in PD subjects and controls, Price et al. [36] found that lower prefrontal fractional anisotropy and caudate nucleus volume were related to reduced processing speed in PD subjects. Thus, dopaminergic dysfunction in networks connecting the striatum and prefrontal cortex and caudate volume loss and dysfunction may be involved in the lower processing speed observed in PD [35, 37–39].

Identification of processing speed deficits is particularly important since this construct has the potential to be modified with cognitive training [40–46]. Edwards et al. [41] demonstrated that PD subjects could improve cognitive processing speed over a three-month period through a self-administered task using speed of processing training (SOPT) software (InSight). Moreover, Milman et al. [47] enrolled 18 PD subjects in a 12-week, computerized cognitive remediation program and found that subjects improved in both gait turning speed and duration. Additional studies are needed to replicate these results and determine whether such cognitive training methods could translate to improved gait, turning, balance, or other activities of daily living (ADLs) in PD.

This study also demonstrated that pace-related measures of gait and postural sway were correlated with global cognitive scores. We were unable to determine the cognitive domain(s) driving this association, likely due to our small sample size. However, prior studies do provide some insight into the cognitive basis for dysfunction in the pace and postural sway domains in PD. Amboni et al. [48] conducted a study in 43 PD subjects and 20 healthy controls that examined the associations between cognition and motor function using an automated sensor-based system generating eight measures categorized into two domains: (1) gait pace and (2) postural stability or balance. Results showed that reduced visuospatial processing was associated with worse postural stability and supported previous studies that describe the importance of processing of visual information for locomotion and motor planning, particularly in PD [49, 50].

In addition to visuospatial function, working memory may also play a role in postural stability. In a cohort of 121 early PD subjects and 184 healthy controls, Lord et al. [51] examined the associations between cognition and motor function using an instrumented walkway system (GAITRite, CIR systems, USA) that generated 16 different gait and balance measures. Results showed a significant association between postural stability and working memory in PD subjects with the PIGD subtype. It was hypothesized that changes in postural stability may be a marker of amnestic features, and that reduced working memory may reflect cortical amyloid deposition in the PIGD subtype of PD [51, 52].

Studies have also suggested a possible relationship between the domain of gait pace and attention [51, 53, 54]. Gait is a goal directed activity, and the finding that attentional circuits are activated through dopaminergic and cholinergic signaling between the dorsolateral prefrontal cortex and the caudate nucleus during gait performance [55] supports this hypothesis. In early PD, individuals may be able to compensate for pace deficits by relying more heavily on these attentional circuits [56]. However as the disease progresses and attentional circuitry becomes increasingly abnormal [57], PD patients are likely less able to compensate [56] and have reduced pace as a result [51].

The strengths of our study include the use of quantitative, sensor-based assessments to evaluate gait and balance that provided objective metrics of motor function, as well as a neuropsychological testing protocol that included global and domain specific cognitive measures. The limitations of our study include a small sample size and the use of only one neuropsychological test per cognitive domain. We also did not include a control group, which would have been helpful in determining which relationships are directly related to PD compared with normal aging. Despite these limitations, our findings highlight the value of linking quantitative motor assessments with clinical neuropsychological measures to elucidate the complex interplay between motor and cognitive function in PD. Overall, this study demonstrates that there is a relationship between specific cognitive domains and motor function in PD which warrants further exploration. We showed that impaired turning is associated with reduced processing speed and larger studies are needed to validate these findings. The question of whether cognitive deficits mediate gait and balance dysfunction or vice versa, or whether there is an interaction between the two processes is an area of active research investigation in PD and other neurodegenerative disorders [7].

## Conclusions

This is the first study to demonstrate the correlation between reduced processing speed and impaired turning in Parkinson’s disease. Identification of processing speed deficits is particularly important since this construct has the potential to be modified with cognitive training. Further studies are needed to validate these findings.

## Appendix

**Table 5:** Gait and balance domains with the corresponding instrumented measures [11]

Instrumented Measure	Unit of Measure	Definition
Gait–pace		Pace during straight ahead walking
Gait cycle time	s	Duration of a complete gait cycle
Cadence	steps/min	Stepping rate
Stride velocity	%h/s	Average gait speed normalized for height
Stride length	%h	Distance between 2 consecutive heel-strikes normalized for height
RoM leg	°	Range of motion (RoM) of the leg (calculated from the integrated sagittal angular velocity, approximation of step length) Average of the left and right sides
Gait–arm & trunk movement		Arm and trunk movement during straight-ahead walking
Arm peak velocity	°/s	Peak (95 %) angular velocity of most affected arm
Arm swing RoM	°	Range of motion of most affected arm during arm swing
Asymmetry arm swing RoM	%	Average asymmetry of left and right arm swing range of motion
RoM trunk frontal plane	°	Average range of motion of trunk in frontal plane
RoM trunk sagittal plane	°	Average range of motion of trunk in sagittal plane
RoM trunk horizontal plane	°	Average range of motion of trunk in horizontal plane
Gait–dynamic stability		Dynamic stability during straight-ahead walking
Double support time	% of gait cycle	Percentage of a gait cycle that both feet are on the ground
Stance time	% of gait cycle	Average percentage of a gait cycle that either foot is on the ground
Swing time	% of gait cycle	Average percentage of a gait cycle that either foot is off the ground
Turning		180° turn
Peak velocity	°/s	Peak (95 %) angular velocity of trunk during turning
Duration	s	Duration of a 180° turn
Mean step time	s	Average duration of step during 180° turn
Number of steps	n	Total number of steps during 180° turn
Postural sway		Standing quietly for 30 s
Sway RMS AP	m/s2	Root mean square (RMS) of acceleration time series in anteroposterior (AP) direction
Sway RMS ML	m/s2	Root mean square of acceleration time series in mediolateral (ML) direction
Mean velocity AP	m/s	Mean velocity of center of pressure in anteroposterior direction
Mean velocity ML	m/s	Mean velocity of center of pressure in mediolateral direction
Centroidal frequency AP	Hz	Centroidal frequency in anteroposterior direction; variability of the acceleration traces power ranging from 0 to 1
Centroidal frequency ML	Hz	Centroidal frequency in mediolateral direction; variability of the acceleration traces power ranging from 0 to 1

Nomenclature^*a*^ ° degree, *g* acceleration of gravity, *%h* percentage of patient’s height, *n* number, *m/s2* acceleration, *s* seconds, − dimensionless

## References

[1] Verghese J (2006). Epidemiology of gait disorders in community-residing older adults. J Am Geriatr Soc.

[2] Srikanth V (2009). Cerebral white matter lesions, gait, and the risk of incident falls: a prospective population-based study. Stroke.

[3] Amar K (2015). Fall frequency, predicting falls and participating in falls research: similarities among people with Parkinson's disease with and without cognitive impairment. Parkinsonism Relat Disord.

[4] Hausdorff JM (2005). Walking is more like catching than tapping: gait in the elderly as a complex cognitive task. Exp Brain Res.

[5] Fling BW (2016). Associations between mobility, cognition and callosal integrity in people with parkinsonism. Neuroimage Clin.

[6] Segev-Jacubovski O (2011). The interplay between gait, falls and cognition: can cognitive therapy reduce fall risk?. Expert Rev Neurother.

[7] Morris R (2016). Gait and cognition: Mapping the global and discrete relationships in ageing and neurodegenerative disease. Neurosci Biobehav Rev.

[8] Recent developments in Parkinson's disease. Edited by S. Fahn, C. D. Mardsen, P. Jenner, and P. Teychenne New York, Raven Press, 1986 375 pp, illustarted. Ann Neurol. 1987;22(5):672.

[9] Kelly VE (2015). Association of cognitive domains with postural instability/gait disturbance in Parkinson's disease. Parkinsonism Relat Disord.

[10] Pal G, Goetz CG (2013). Assessing bradykinesia in parkinsonian disorders. Front Neurol.

[11] Mancini M (2015). Continuous monitoring of turning in Parkinson's disease: Rehabilitation potential. Neuro Rehabilitation.

[12] Bonora G (2015). A new instrumented method for the evaluation of gait initiation and step climbing based on inertial sensors: a pilot application in Parkinson's disease. J Neuroeng Rehabil.

[13] Horak FB, Mancini M (2013). Objective biomarkers of balance and gait for Parkinson's disease using body-worn sensors. Mov Disord.

[14] Del Din S (2016). Free-living gait characteristics in ageing and Parkinson’s disease: impact of environment and ambulatory bout length. J. NeuroEng. Rehabil..

[15] Mancini M (2009). Anticipatory postural adjustments prior to step initiation are hypometric in untreated Parkinson's disease: an accelerometer-based approach. Eur J Neurol.

[16] Salarian A (2009). Analyzing 180 degrees turns using an inertial system reveals early signs of progression of Parkinson's disease. Conf Proc IEEE Eng Med Biol Soc.

[17] Salarian A (2004). Gait assessment in Parkinson's disease: toward an ambulatory system for long-term monitoring. IEEE Trans Biomed Eng.

[18] Hughes AJ (1992). Accuracy of clinical diagnosis of idiopathic Parkinson's disease: a clinico-pathological study of 100 cases. J Neurol Neurosurg Psychiatry.

[19] Hoehn MM, Yahr MD (1967). Parkinsonism: onset, progression and mortality. Neurology.

[20] Jankovic J (1990). Variable expression of Parkinson's disease: a base-line analysis of the DATATOP cohort. The Parkinson Study Group. Neurology.

[21] Horak FB (2016). Balance and Gait Represent Independent Domains of Mobility in Parkinson Disease. Phys Ther.

[22] Dewey DC (2014). Automated gait and balance parameters diagnose and correlate with severity in Parkinson disease. J Neurol Sci.

[23] Curtze C (2015). Levodopa Is a Double-Edged Sword for Balance and Gait in People With Parkinson's Disease. Mov Disord.

[24] Mattis S (1976). Mental Status examination for organic mental syndrome in the elderly patient.

[25] Mattis S (1988). Dementia Rating Scale Professional Manual.

[26] Glickman ME, Rao SR, Schultz MR (2014). False discovery rate control is a recommended alternative to Bonferroni-type adjustments in health studies. J Clin Epidemiol.

[27] Curtze C, et al. Objective Gait and Balance Impairments Relate to Balance Confidence and Perceived Mobility in People With Parkinson Disease. Phys Ther. 2016. 10.2522/ptj.20150662 PMC5088223 27149959

[28] Pelosin E (2016). Attentional Control of Gait and Falls: Is Cholinergic Dysfunction a Common Substrate in the Elderly and Parkinson's Disease?. Front Aging Neurosci.

[29] Maidan I (2016). Altered brain activation in complex walking conditions in patients with Parkinson's disease. Parkinsonism Relat Disord.

[30] Stuart S (2016). Gait in Parkinson's disease: A visuo-cognitive challenge. Neurosci Biobehav Rev.

[31] Salarian A (2010). iTUG, a sensitive and reliable measure of mobility. IEEE Trans Neural Syst Rehabil Eng.

[32] Van Uem JM (2016). Quantitative Timed-Up-and-Go Parameters in Relation to Cognitive Parameters and Health-Related Quality of Life in Mild-to-Moderate Parkinson's Disease. PLoS One.

[33] van der Wardt V (2015). The Association of Specific Executive Functions and Falls Risk in People with Mild Cognitive Impairment and Early-Stage Dementia. Dement Geriatr Cogn Disord.

[34] Albinet CT (2012). Processing speed and executive functions in cognitive aging: how to disentangle their mutual relationship?. Brain Cogn.

[35] Jokinen P (2013). Cognitive slowing in Parkinson's disease is related to frontostriatal dopaminergic dysfunction. J Neurol Sci.

[36] Price CC (2016). Gray and White Matter Contributions to Cognitive Frontostriatal Deficits in Non-Demented Parkinson's Disease. PLoS One.

[37] Marklund P (2009). Temporal dynamics of basal ganglia under-recruitment in Parkinson's disease: transient caudate abnormalities during updating of working memory. Brain.

[38] Bohnen NI, Martin WR (2014). Dopamine-dependent functional connectivity in Parkinson disease: a resting-state diagnosis?. Neurology.

[39] Helie S, Cousineau D (2015). Differential effect of visual masking in perceptual categorization. J Exp Psychol Hum Percept Perform.

[40] Cerasa A (2014). Neurofunctional correlates of attention rehabilitation in Parkinson's disease: an explorative study. Neurol Sci.

[41] Edwards JD (2013). Randomized trial of cognitive speed of processing training in Parkinson disease. Neurology.

[42] Pena J (2014). Improving functional disability and cognition in Parkinson disease: randomized controlled trial. Neurology.

[43] Paris AP (2011). Blind randomized controlled study of the efficacy of cognitive training in Parkinson's disease. Mov Disord.

[44] Petrelli A (2014). Effects of cognitive training in Parkinson's disease: a randomized controlled trial. Parkinsonism Relat Disord.

[45] Pompeu JE (2012). Effect of Nintendo Wii-based motor and cognitive training on activities of daily living in patients with Parkinson's disease: a randomised clinical trial. Physiotherapy.

[46] Sammer G (2006). Training of executive functions in Parkinson's disease. J Neurol Sci.

[47] Milman U (2014). Can cognitive remediation improve mobility in patients with Parkinson's disease? Findings from a 12 week pilot study. J Parkinsons Dis.

[48] Amboni M (2012). Gait patterns in Parkinsonian patients with or without mild cognitive impairment. Mov Disord.

[49] Helmich RC (2007). Cerebral compensation during motor imagery in Parkinson's disease. Neuropsychologia.

[50] Lewis GN, Byblow WD, Walt SE (2000). Stride length regulation in Parkinson's disease: the use of extrinsic, visual cues. Brain.

[51] Lord S (2014). Cognition and gait show a selective pattern of association dominated by phenotype in incident Parkinson's disease. Front Aging Neurosci.

[52] Alves G (2013). Cerebrospinal fluid amyloid-beta and phenotypic heterogeneity in de novo Parkinson's disease. J Neurol Neurosurg Psychiatry.

[53] Lord S (2010). Executive dysfunction and attention contribute to gait interference in 'off' state Parkinson's Disease. Gait Posture.

[54] Rochester L (2008). Walking speed during single and dual tasks in Parkinson's disease: which characteristics are important?. Mov Disord.

[55] Calabresi P (2006). A convergent model for cognitive dysfunctions in Parkinson's disease: the critical dopamine-acetylcholine synaptic balance. Lancet Neurol.

[56] Redgrave P (2010). Goal-directed and habitual control in the basal ganglia: implications for Parkinson's disease. Nat Rev Neurosci.

[57] Chahine LM (2016). Longitudinal changes in cognition in early Parkinson's disease patients with REM sleep behavior disorder. Parkinsonism Relat Disord.

